# Supplementary material for: Deaths from undetected COVID-19 infections as a fraction of COVID-19 deaths can be used for early detection of an upcoming epidemic wave
Source: PLoS One. 2023 Mar 17;18(3):e0283081. doi: 10.1371/journal.pone.0283081 (PMC10022783; doi:10.1371/journal.pone.0283081)
Supplement: Attachment — Submitted filename: Response to reviewers.pdf [file pone.0283081.s001.pdf]

Responses to reviewers

We thank the reviewers for their valuable feedback and suggestions. Their constructive critique of our manuscript has positively motivated us to incorporate finer details into this work. In this document we have listed the reviewers' comments in ***Bold Italics*** and have answered all their concerns inline with a blue text. References are listed below and cited within [square brackets highlighted in yellow]

Reviewer #1: Thank you for providing me with an opportunity to review this article. I appreciate the authors for working on an important topic. The authors have proposed an indicator as a measure to estimate the extent of latent infection in populations. There is some ambiguity with the study.
Q1: for example, although the authors have targeted the COVID-19 pandemic, in the method section the parameters are presented generally and not specified to COVID-19. Moreover, the authors' interpretation seems as if the formulas and parameters used in the model can be used in any pandemic, while every pandemic might have a different pattern undermining the proposed parameters.

Thank you very much for your comment. Some key parameters used to model COVID 19 epidemic as seen in [1,2,3] are

1. Reproduction number R0 - mean number of infections an infected person might cause
2. Serial Interval (mean infection time) - The time between one infected individual infecting another healthy individual.
3. Incubation period (mean latency time) - the time between infection and onset of disease
4. Transmissibility period - time during which an infected person transmits the virus to other individuals.
5. Proportion of confirmed cases in the population
6. Proportion of deaths in critical cases etc.
7. Beta

We have used an epidemiological simulator from [4] that defines most of these parameters as mentioned in the table below.

Table 1

Parameter	Value

inf_t - mean infection time	9
lat_t - mean latency time	7
in_phi - rate of population inflow	5
pI - probability of infection in the population inflow	0.1
Number of days predicted by the simulator	125
Number of days simulated before the 1st infection	50
Population Size	10 000 000
a - fraction of infections through contact	0.3
β	0.35

These parameters are tunable and hence can be used for any epidemic where their values might be different. We have configured it to fit the covid19 data obtained from https://covid19tracker.in/. Some biological parameters like duration of immunity post recovery, change in morbidity post reinfections etc can be analyzed through sero-surveys but the data for that is sparsely sampled and not easily available. Further the transmissibility of infection is different in different age groups and in asymptomatic individuals and there can also be some unknown pathways for the spread of infections[2]. We have not modeled these parameters owing to their limited access.

Addressing this comment we have added these points in the Introduction section

Q2: It seems that there is a difference between the modeling estimates and real-world results in the study. I kindly recommend the authors compare the real data (COVID-19 data) with simulation results in their modeling study to investigate the accuracy of the simulation. For example, in their figures, the authors have estimated the pandemic with a single peak in 200 days; whereas in the real world, we have seen several peaks within such period. And each peak has different parameter quantities.

Thank you very much for your valuable feedback. We have incorporated your comments in our simulation of the real world data of covid 19 in India and have presented the results in the

“Performance of D_ratio and other metrics on real world data” subsection. This helped us validate our hypothesis on a larger real world data.

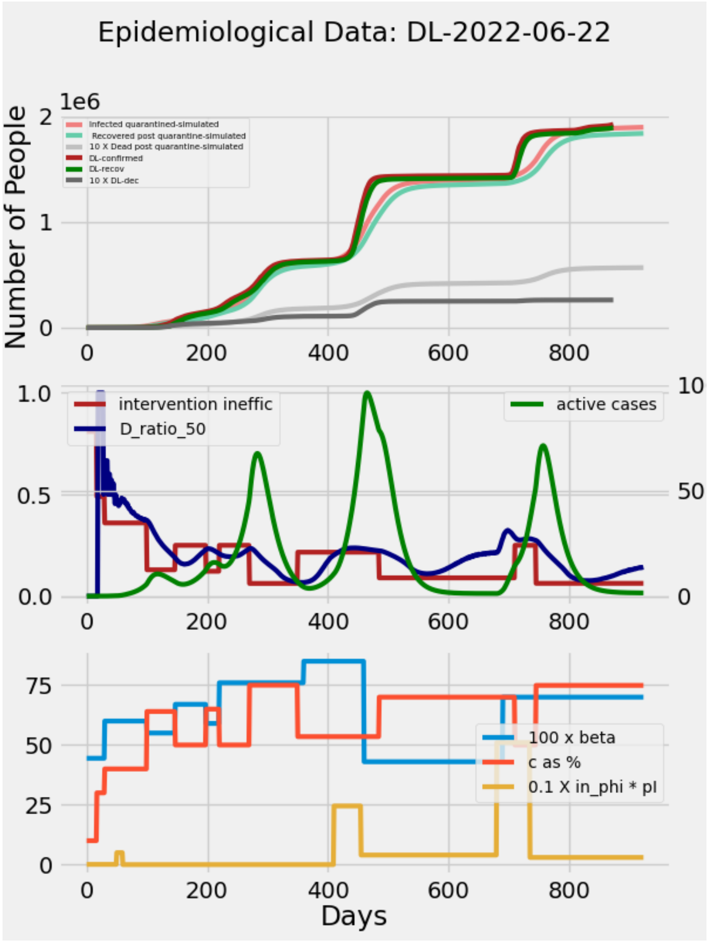

Figure 1: Delhi as on 22nd June 2022. Top to bottom: (a) Real covid19 data and simulated cases on natural scale are plotted and it can be observed that the simulations match the real cases to a good extent. (b) The intervention inefficiency underlying the epidemic is plotted along with D_ratio metric and Active cases. It is evident from the figure that D_ratio also distinctly identifies the 3 waves of the epidemic and also that it lags the active cases metric by 36 days. (c) The plot describes how the free variables of the model are tuned in the course of the epidemic to fit the model to real data. It is seen that before every wave, there is an increase in influx of migrant population and beta while there is a decrease in c.

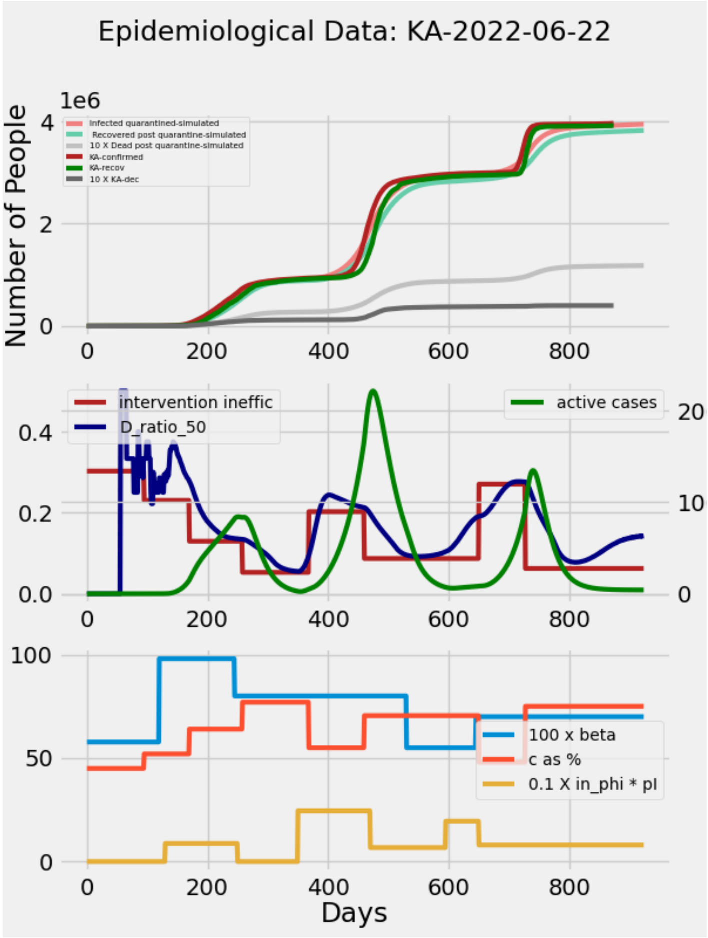

Figure 3: Karnataka as on 22nd June 2022. Top to bottom : (a) Real covid19 cases and simulated cases. (b) D_ratio lags active cases by 58 days. (c) Free variables modified to fit the epidemic

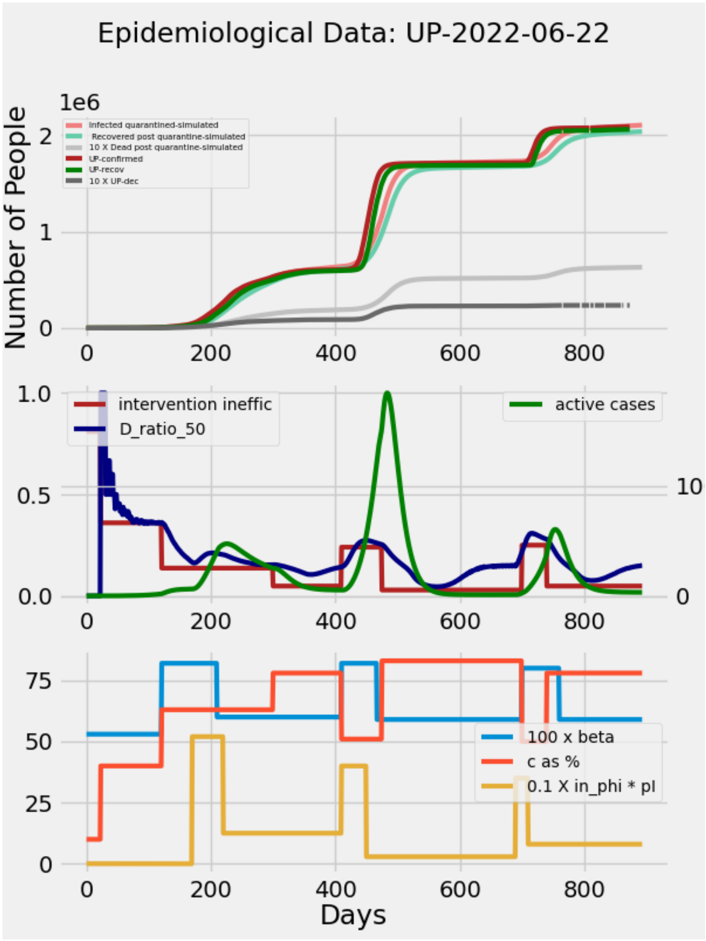

Figure 3: Uttar Pradesh as on 22nd June 2022. Top to bottom : (a) Real covid19 cases and simulated cases. (b) D_ratio lags active cases by 31 days. (c) Free variables modified to fit the epidemic

We fit our model to three Indian states from the period of Jan 1 2020 to June 06 2022. The model fitting is achieved by tuning the free variables: influx of migrant population, probability of infections from migrant population , beta and percentage of contact tracing parameter c. Further a cross correlation is performed on the metrics D_ratio and active cases to identify the similarity and time lag between the two metrics of interest. It is observed that a wave is precipitated when beta and influx of migrant population increases and c decreases significantly. As demonstrated in Fig~[1,2,3] the metric D_ratio always lags the metric active cases by a good number of days thereby validating its utility in the purpose of planning mitigation strategies. Even the time delay from cross correlation between the two metrics of interest corroborates the same. Comparison of daily new cases to D_ratio showed that daily new cases are extremely noisy and give more

false positive waves. Hence daily new cases don't surface as a reliable measure to predict waves. These results prove the advantages of a metric like D_ratio in real world scenarios giving the administrative bodies close to 25-30 days to prepare for an upcoming wave and also to bring in interventions by keeping a tab on the underlying model parameters.

Q3: Also, it is not clear how the authors have calculated the number of undetected cases (D) and what is their rational to do so in different types of pandemics.

Undetected cases in our model are obtained from simulations. Undetected cases are typically estimated from techniques like PCR and antibody tests.[5] describes how there are very few strategies to identify the number of undetected cases in the population, most popular ones being PCR and antibody tests. These tests sample the population randomly and only estimate the undetected cases in a specific population, at a specific time point. Both these techniques are very sparsely sampled, forcing us to fallback on simulations for estimating the undetected cases in the population. We have estimated the populations of each compartment of the epidemic in the SEIRD model defined by the differential equations in [4]. In our experiment we have fit the model to actual covid19 data in different Indian states, thereby matching the simulated cases to confirmed cases, recoveries and deaths, hence making our estimate of undetected cases also realistic in a fixed population.

We use the undetected cases to predict an epidemic wave much earlier than other known metrics like active cases or reproductive numbers. Hospitalizations and deaths are unmissable events in an epidemic and are better indicators of growth in an epidemic [6]. We use this information to derive the D_ratio metric that can foresee an epidemic wave.

We have addressed this comment in the introduction section.

Q4: More importantly, we use any indicator for a specific purpose. However, in the current study, it is not clear for what specific purpose the proposed indicator should be used. Comparing the different indicators depends on the purpose.

Popular indicators used to analyze the case growth are Reproductive number, active cases, daily new cases and test positivity rates. [7] Analyses the case growth pattern in Indian states

by clustering and ranking them based on severity using metrics like number of active cases and death rates.But this technique needs enough data, and hence a considerable time into the epidemic would have elapsed by the time we get some insights. [8] discusses the correlation between reproductive rate(Rt) and Test positivity rates in different parts of the world. The authors show that a strong positive correlation between Test positivity rate and Rt in India indicates that testing was insufficient to catch up with the increasing cases. Even though test positivity rate correlates with Reproductive number R, and indicates an impending wave, it is highly biased towards the testing strategies in different places (different states) and the cooperation by the population. This induces variance in the measure making it a little unreliable. Further contact tracing is also biased to the serial interval in symptomatic people [2] .Hence there is a need for a new metric that overcomes these shortcomings and predicts a wave much earlier than the described ones. We propose that D_ratio is one such metric that is not sensitive to testing strategies and can be estimated from simulations that gives us sufficient time to plan mitigation measures.

Addressing this comment, we have added these points in the introduction section.

Q5: I also suggest the authors strengthen their paper by using a few more references and discussing their results, comparing them with other studies in similar contexts.

We have added the below references in addition to the existing references.

1. Kwuimy CAK, Nazari F, Jiao X, Rohani P, Nataraj C. Nonlinear dynamic analysis of an epidemiological model for COVID-19 including public behavior and government action. Nonlinear Dyn [Internet]. 2020;101(3):1545–59. Available from: http://dx.doi.org/10.1007/s11071-020-05815-z

2. Reproduction number (R) and growth rate (r) of the COVID-19 epidemic in the UK: methods of estimation, data sources, causes of heterogeneity, and use as a guide in policy formulation [Internet]. Royalsociety.org. 2020 [cited 2022 Jul 14]. Available from: https://royalsociety.org/-/media/policy/projects/set-c/set-covid-19-R-estimates.pdf?la=en-GB&hash=FDFFC11968E5D247D8FF641930680BD6

3. Gallo LG, Oliveira AF de M, Abrahão AA, Sandoval LAM, Martins YRA, Almirón M, et al. Ten epidemiological parameters of COVID-19: Use of rapid literature review to inform predictive models during the pandemic. Front Public Health [Internet]. 2020;8:598547. Available from:http://dx.doi.org/10.3389/fpubh.2020.598547

4. Rippinger C, Bicher M, Urach C, Brunmeir D, Weibrecht N, Zauner G, et al. Evaluation of undetected cases during the COVID-19 epidemic in Austria. BMC Infect Dis [Internet]. 2021;21(1):70. Available from: http://dx.doi.org/10.1186/s12879-020-05737-6

5. Holmdahl I, Buckee C. Wrong but useful - what covid-19 epidemiologic models can and cannot tell us. N Engl J Med [Internet]. 2020;383(4):303–5. Available from: http://dx.doi.org/10.1056/NEJMp2016822

6. Raj A, Bhattacharyya P, Gupta GR. Clusters of COVID-19 indicators in India: Characterization, correspondence and change analysis. SN Comput Sci [Internet]. 2022;3(3):210. Available from: http://dx.doi.org/10.1007/s42979-022-01083-3

7. Furuse Y, Ko YK, Ninomiya K, Suzuki M, Oshitani H. Relationship of test positivity rates with COVID-19 epidemic dynamics. Int J Environ Res Public Health [Internet]. 2021 [cited 2022 Jul 15];18(9). Available from: http://dx.doi.org/10.3390/ijerph18094655

8. Jones A, Strigul N. Is spread of COVID-19 a chaotic epidemic? Chaos Solitons Fractals [Internet]. 2021 [cited 2022 Jul 15];142(110376):110376. Available from: https://pubmed.ncbi.nlm.nih.gov/33100605/

Reviewer #2: [1] This article uses a modified SEIRD model to show that the ratio of deaths and hospitalizations from an undetected infection to total deaths converges to a measure of systemic test-trace inefficiency. I think there is enough new content in this paper to distinguish it from other works, considering that in recent years many articles have been published on the use of mathematical models for the dynamics of the COVID-19 pandemic, but without any practical utility.
Q6: However, before proposing that policy makers and health administrators should consider including the Dratio metric as part of their decision-making framework, it is necessary that this paper is not merely a mathematical application of a model but also considers important biological assumptions.

Thank you very much for your constructive feedback. We have incorporated your suggestions in Introduction and Discussion sections as well as in the answer below. The covid19 pandemic is also characterized by many biological parameters like transmissibility across age groups, The effect of vaccination, and different epidemiological parameters in different strains of the virus etc. But most of these biological parameters are not easy to study in an ongoing epidemic [6]. These parameters are obtained largely from sero-surveys, virological testing which are sparsely sampled. Models that are fit to confirmed cases are sensitive to testing strategies. Hence the models fit to deaths and hospitalizations are more reliable [6] . Our model doesn't account for the immunological changes, transmissibility changes due to reinfections [2], vaccination and

mutations of viral strains. Further we have enlisted the model assumptions and limitations in (Q 8) in detail.

Q7: In a recent article, Jones and Strigul (2021) hypothesized that the unpredictability of the COVID-19 pandemic could be a fundamental property if the disease spread is a chaotic dynamical system. This means that the change in daily numbers of COVID-19 is affected by a very large number of factors, such as the population's adherence to prevention measures, vaccination, social isolation, and new variants of the virus. In addition, in a recent letter published in the Journal of Medical Virology, Divino and colleagues advert that models used to generate predictions, scenarios, and projections about COVID-19 infections and hospitalizations, are unreliable (https://doi.org/10.1002/jmv.27325).

Jones and Strigul (2021)[9] have compared the growth in different countries with different underlying parameters resulting in a chaotic dynamical system like behavior. But in this work we have set out to provide the administrative bodies an aid to make specific reforms suited for curtailing the growth of epidemic in a particular state where there is less variance in the underlying parameters. The model parameters beta,c,q and infections from influx of migrant population are considered as piecewise constant values. Also since all these parameters are inferred from the same field data, we believe that unpredictability due to chaotic dynamical systems affects both the Death ratios and active cases equally. Hence we can still use the metric regardless of the chaotic behavior. From the results in the paper, we see that the model is fit to real world data in 3 different Indian states highlighting that the model is configurable to a specific region and uses actual field data to analyze the underlying parameters unlike a general set of models that Divino and colleagues describe in their letter[10]. Further [6] states that deaths and hospitalization data are more reliable compared to projections made on the basis of reproductive rates. Hence we demonstrate the utility of a metric derived from the number of deaths in a population to predict a wave.

We have addressed these comments in the Discussions section

Q8: Therefore, the authors must describe the scope and potential limitations of the proposed model from a clinical and epidemiological, not only mathematical, perspective, justifying how these exogenous variables can affect the results obtained.

1. Following an SEIRD model, our model assumes that a person infected once will not be re-infected.

2. Localized effects of change in the dynamics are not captured by the model. It means that there could be a denser spread of cases in certain districts while sparser cases in most other districts.
3. Changing mortality rates with vaccinations, strains etc are not accounted for in the model. The effect of changing mortality is visible in the third wave that can be seen in the error between the predicted number of cases and the observed number of cases when fitted to real world data. Despite this discrepancy we observe that the utility of the metric D_ratio is still valid.

Addressing the comment, we have added these points in the Discussion section.

Q9: Future perspectives for more complex models, including other factors, are also critical.

Our model assumes a constant fatality rate throughout the epidemic. However we have seen that the mortality rates have been different in each wave and different for each strain of the virus. Hence the model can be improved by parameterizing different strains of the virus,taking into account the changing mortality rates and the effects of vaccination and the resultant changes in transmissibility in each individual.The model can also be designed to account for reinfections. Transmissibility within symptomatic and asymptomatic people can be different and can be parameterized in the model to improve the accuracy of predictions.Incorporating all these clinical and biological parameters into the model will surely make it extensive and more accurate. But in the case of epidemics caused by a completely new pathogen, the cases would surge rapidly affecting the greater population by the time the nature of the pathogen is completely understood. We show that a simple model with the mentioned parameters is just enough to capture the metric D_ratio that serves as an early indicator of the impending wave. We believe that this metric empowers the administration and hospitals to introduce timely interventions, plan and strategize better resource allocation.

Addressing the comment, we have added these points in the Discussion section.

Reference: Jones A, Strigul N. Is spread of COVID-19 a chaotic epidemic? Chaos Solitons Fractals. 2021;142:110376.

Q10: Please justify the parameters shown in Table 1.

We arrived at the seed parameters from the covid19 data till 27th April 2020 to ensure that the determined parameters are epidemiologically plausible. It was earlier estimated that mean infection time of covid 19 was 9-10 days and mean latency period was 1 week. Population size of 10 million is chosen to keep it realistic to the population of an Indian city. The other parameters like beta and influx of migrant population and their probability of spreading infections are considered to be time varying in the course of the epidemic. Starting with these parameters as the seed, these time varying parameters were modified to simulate and validate the purpose of the D_ratio metric in our study. [Table 1]

We have added these details in the methods section of the paper.

Q11: Please provide a sensitivity analysis considering changes in the parameters shown in Table 1.

Thank you very much for your suggestion. We have addressed this comment in the subsection titled ***Sensitivity of D_ratio to model parameters*** in the Results section. This has helped us characterize and describe the model better. From the table [Table 1] we can largely classify the parameters as static parameters that don't change in the course of epidemic simulation and dynamic parameters that are time varying and are manipulated throughout the simulation to fit the model to some particular data. The sensitivity analysis has been performed on these static parameters namely the mean infection time (inf_t), the mean latency time (lat_t), population, and fraction of infections through direct contact (a). It was observed that while the model was *sensitive to mean infection time* and *mean latency period*, *population* and *a* did not affect the model with respect to the metric D_ratio. The sensitivity analysis summary is presented in the figure below [Fig 4].

Time lag between D_ratio and number of active cases						
**lat_t**	**3**	**5**	**7**	**9**	**15**	**30**
**inf_t**						
**6**	27	45	78	86	88	87
**9**	0	23	39	54	96	97
**15**	-25	-2	17	32	71	103
**30**	-25	-12	1	13	48	98

Figure 4 : Table showing the number of days D_ratio lags the number of active cases for different values of inf_t and lat_t. The lag is obtained by performing a cross correlation of D_ratio with the number of active cases.

From this table it can be observed that the D_ratio is not effective when the mean infection times are very high compared to mean latency times and such a case hasn't been encountered so far.

REFERENCES:

1. Kwuimy CAK, Nazari F, Jiao X, Rohani P, Nataraj C. Nonlinear dynamic analysis of an epidemiological model for COVID-19 including public behavior and government action. Nonlinear Dyn [Internet]. 2020;101(3):1545–59. Available from: http://dx.doi.org/10.1007/s11071-020-05815-z

2. Reproduction number (R) and growth rate (r) of the COVID-19 epidemic in the UK: methods of estimation, data sources, causes of heterogeneity, and use as a guide in policy formulation [Internet]. Royalsociety.org. 2020 [cited 2022 Jul 14]. Available from: https://royalsociety.org/-/media/policy/projects/set-c/set-covid-19-R-estimates.pdf?la=en-GB&hash=FDFFC11968E5D247D8FF641930680BD6

3. Gallo LG, Oliveira AF de M, Abrahão AA, Sandoval LAM, Martins YRA, Almirón M, et al. Ten epidemiological parameters of COVID-19: Use of rapid literature review to inform predictive models during the pandemic. Front Public Health [Internet]. 2020;8:598547. Available from:http://dx.doi.org/10.3389/fpubh.2020.598547

4. Raghavan M, Sridharan KS, Mandayam Rangayyan Y. Using epidemic simulators for monitoring an ongoing epidemic. Sci Rep [Internet]. 2020 [cited 2022 Jul 15];10(1):16571. Available from: https://www.nature.com/articles/s41598-020-73308-5

5. Rippinger C, Bicher M, Urach C, Brunmeir D, Weibrecht N, Zauner G, et al. Evaluation of undetected cases during the COVID-19 epidemic in Austria. BMC Infect Dis [Internet]. 2021;21(1):70. Available from: http://dx.doi.org/10.1186/s12879-020-05737-6

6. Holmdahl I, Buckee C. Wrong but useful - what covid-19 epidemiologic models can and cannot tell us. N Engl J Med [Internet]. 2020;383(4):303–5. Available from: http://dx.doi.org/10.1056/NEJMp2016822

7. Raj A, Bhattacharyya P, Gupta GR. Clusters of COVID-19 indicators in India: Characterization, correspondence and change analysis. SN Comput Sci [Internet]. 2022;3(3):210. Available from: http://dx.doi.org/10.1007/s42979-022-01083-3

8. Furuse Y, Ko YK, Ninomiya K, Suzuki M, Oshitani H. Relationship of test positivity rates with COVID-19 epidemic dynamics. Int J Environ Res Public Health [Internet]. 2021 [cited 2022 Jul 15];18(9). Available from: http://dx.doi.org/10.3390/ijerph18094655

9. Jones A, Strigul N. Is spread of COVID-19 a chaotic epidemic? Chaos Solitons Fractals [Internet]. 2021 [cited 2022 Jul 15];142(110376):110376. Available from: https://pubmed.ncbi.nlm.nih.gov/33100605/

10. Divino F, Ciccozzi M, Farcomeni A, Jona-Lasinio G, Lovison G, Maruotti A. Unreliable predictions about COVID-19 infections and hospitalizations make people worry: The case of Italy. J Med Virol [Internet]. 2022;94(1):26–8. Available from: http://dx.doi.org/10.1002/jmv.27325
