# Supplementary material for: Deaths from undetected COVID-19 infections as a fraction of COVID-19 deaths can be used for early detection of an upcoming epidemic wave
Source: PLoS One. 2023 Mar 17;18(3):e0283081. doi: 10.1371/journal.pone.0283081 (PMC10022783; doi:10.1371/journal.pone.0283081)
Supplement: Attachment — Submitted filename: Response to reviewers.pdf [file pone.0283081.s002.pdf]

Responses to reviewers

We thank the reviewers for their valuable feedback and suggestions. Their constructive critique of our manuscript has positively motivated us to incorporate finer details into this work. In this document we have listed the reviewers' comments in ***Bold Italics*** and have answered all their concerns inline with a blue text. References are listed below and cited within [square brackets highlighted in yellow]

Reviewer #3: OVERVIEW
While it is important to examine the potential for new metrics for monitoring epidemics, as the authors suggest here their D_ratio of deaths to hospitalizations, there is still a lack of clarity. The manuscript Introduction should begin describing the COVID-19 pandemic or at least epidemics, and the manuscript definitely needs further proofreading/editing.

Thank you very much for your feedback. We have incorporated your suggestions and have reorganized the introduction as described in our response to Q2 under Major comments below in this document.

COMMENTS ON THE AUTHORS' RESPONSE TO REVIEWERS

Response to reviewer #1, Q2
In Figure 1, could the authors ensure there are panel labels (a, b, c) rather than top to bottom, the figure caption needs proofreading edits. Perhaps it would be clearer to refer to 'real' COVID-19 data as actual data. The legend text in panel (a) is too small to read. Define the 'D_ratio' on first use in this figure for clarity. No need to capitalize 'Active' cases. No need to include 'it is evident'. Ensure the legend is panel (b) is fully spelled out, i.e., 'intervention ineffc'. In the description for panel (c) what do the authors mean by 'free' variables? Not fixed? Also define 'c' in the figure caption (last character in the response). Between figures 1 and 3 panels (b), can the authors explain why there is a difference in the number of days of lag, i.e., 36 days for Delhi vs 58 days for Karnataka vs 31 days for UP– unless this has been covered in the main manuscript. For Figure 3, the description for panel (c) notes that these free variables were modified to fit the epidemic, but from the figure the fitting is not clear. Please explain or modify. In the response the authors note the model was fit to data from 1 January 2020 to 6 June 2022, but in the figure captions the dates are listed as 22 June 2022, why the difference? Most often new daily cases are reported as a 7-day rolling average, not as the authors note (daily new cases) surrounding the note around noisiness potentially leading to more false positive waves. Again, in the authors' response this method may raise a warning flag, but the message around needing to bring in interventions needs to be placed in the context of pandemic fatigue.

Thank you very much for your suggestions. We have made the necessary changes to the figure caption as below and have also labeled the panels as (a),(b) and (c). We have also tuned the model to fit to real data better and have presented them in the revised manuscript. The error in the date mentioned in our response to reviewers was inadvertent and we have corrected it in the manuscript. We would like to clarify that all the metrics and indicators that we have used for comparison in the manuscript have been reported after performing n-day rolling averages(n=7,10,15,25,50). Acknowledging the review comment, we have now explicitly mentioned this in the revised manuscript once again. As for the comparison of the performance of D_ratio with active cases and daily new cases, we have switched to a more robust method of identifying an appropriate time point for intervention. We look at the day when any metric of interest crosses its maximum value in the last 20 days to make any intervention.

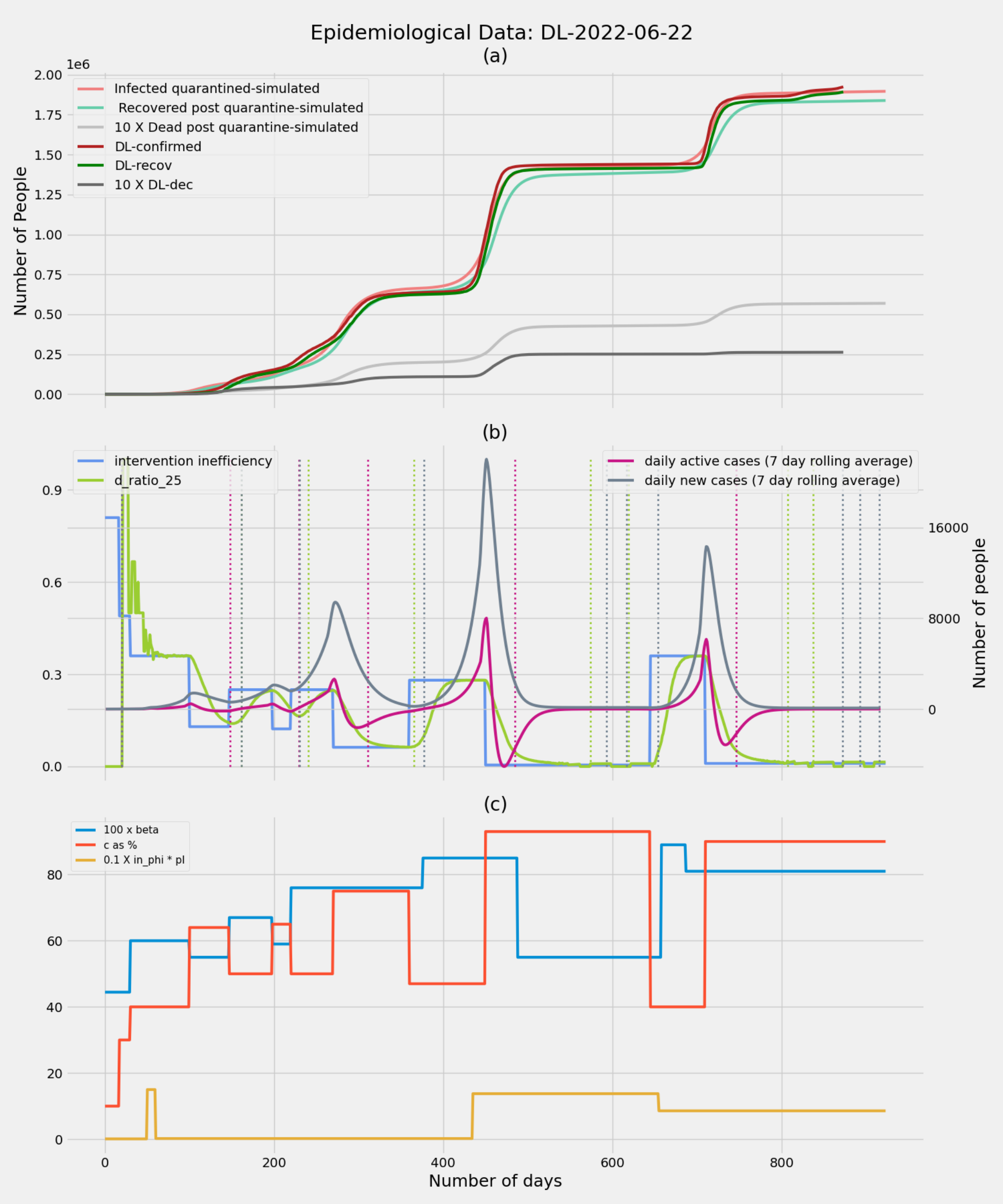

Figure 1 : Delhi as on 22nd June 2022. (a) Real COVID-19 data and simulated data showing confirmed cases, recoveries and deaths. It is observed that simulations match the real case growth to a great extent. (b) The intervention inefficiency underlying the epidemic is plotted along with the ratio of deaths from undetected infections to total number of deaths (D_ratio), daily new cases and active cases. The dashed vertical lines correspond to the potential time points when an intervention can be made based on the respectively coloured D_ratio, daily active cases and daily new cases. D_ratio predicts an upcoming wave ahead of other metrics when the wave is precipitated due to drop in efficiency (decreasing values of 'c' -the fraction of infections detected through contact tracing). (c) The model parameters beta, fraction of infections that get detected through contact tracing - 'c', the fraction of infections spread through migrant population - 'in_phi*pI' are tuned to fit the model to real data.

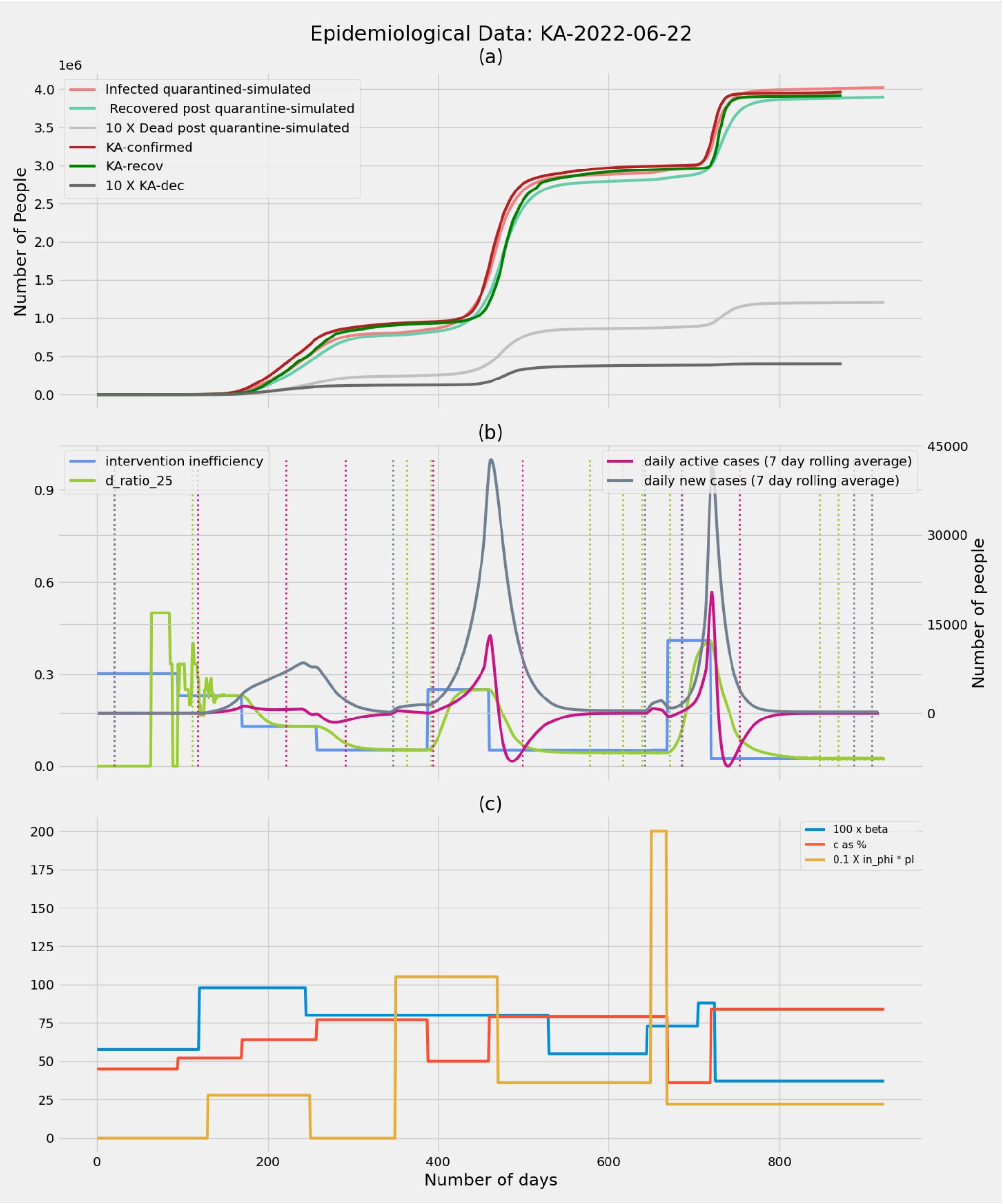

Figure 2 : Karnataka as on 22nd June 2022. (a) Real COVID-19 data and simulated data showing confirmed cases, recoveries and deaths. It is observed that simulations match the real case growth to a great extent. (b) The intervention inefficiency underlying the epidemic is plotted along with the ratio of deaths from undetected infections to total number of deaths (D_ratio), daily new cases and active cases. The dashed vertical lines correspond to the potential time points when an intervention can be made based on the respectively coloured D_ratio, daily active cases and daily new cases. D_ratio predicts an upcoming wave ahead of other metrics when the wave is precipitated due to drop in efficiency (decreasing values of ‘c’ -the fraction of infections detected through contact tracing). (c) The model parameters beta, fraction of infections that get detected through contact tracing - ‘c’, the fraction of infections spread through migrant population - ‘in_phi*pI’ are tuned to fit the model to real data.

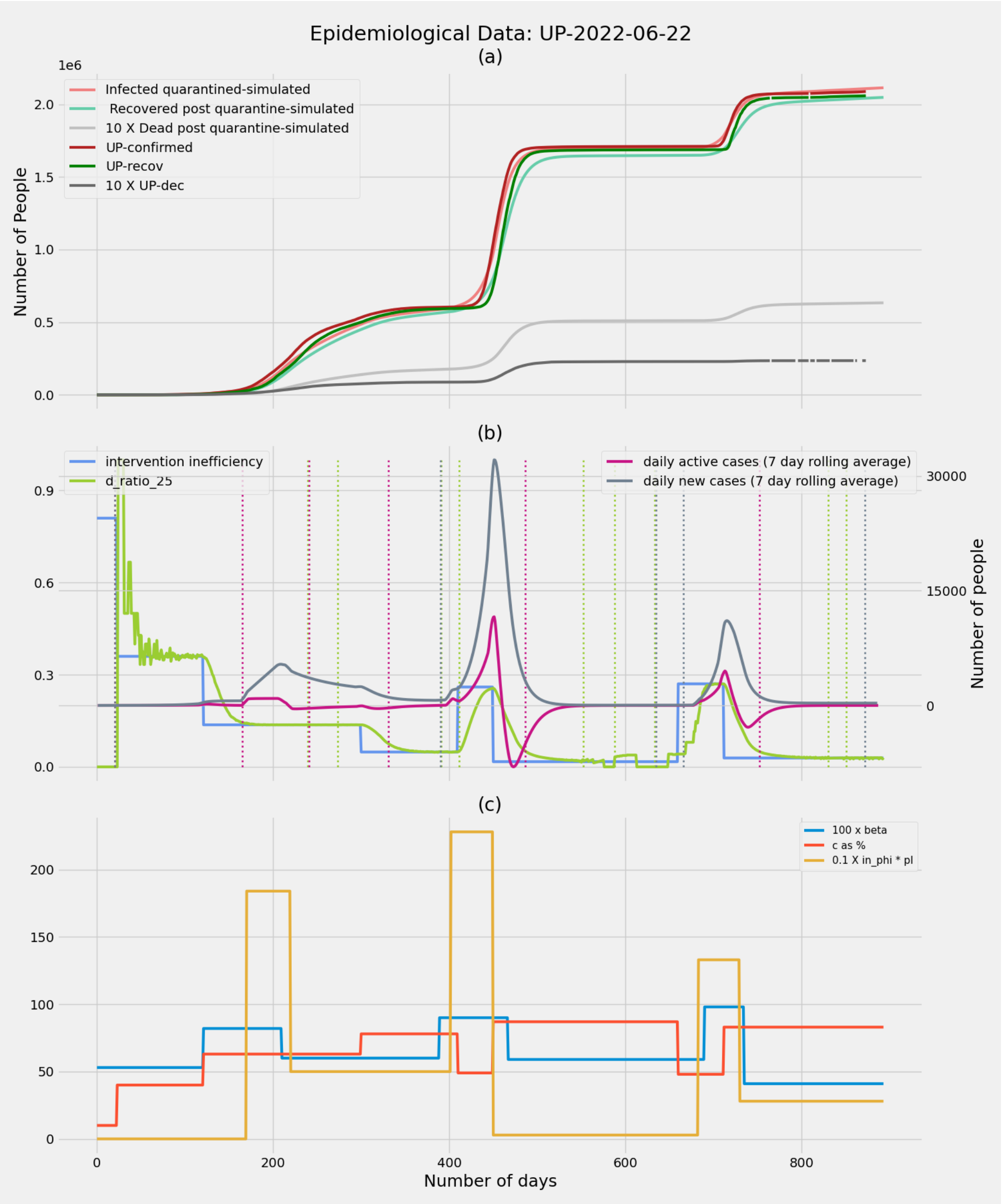

Figure 3 : Uttar Pradesh as on 22nd June 2022. (a) Real COVID-19 data and simulated data showing confirmed cases, recoveries and deaths. It is observed that simulations match the real case growth to a great extent. (b) The intervention inefficiency underlying the epidemic is plotted along with the ratio of deaths from undetected infections to total number of deaths (D_ratio), daily new cases and active cases. The dashed vertical lines correspond to the potential time points when an intervention can be made based on the respectively coloured D_ratio, daily active cases and daily new cases. D_ratio predicts an upcoming wave ahead of other metrics when the wave is precipitated due to drop in efficiency (decreasing values of 'c' -the fraction of infections detected through contact tracing). (c) The model parameters beta, fraction of infections that get detected through contact tracing - 'c', the fraction of infections spread through migrant population - 'in_phi*pI' are tuned to fit the model to real data.

Response to reviewer #1, Q3
Can the authors be very clear that if they fit to confirmed cases, the undetected cases they estimate would be in addition to the confirmed cases that are fit, for a total estimated cases (confirmed + undetected)?

The model starts with an entire population of the state being Susceptible. The dynamics of the epidemic determines the fraction of population in each subsequent compartments of Exposed, Infectious, Recovered and Deceased. When the model is fit to the real data in a state, the model parameters are configured such that the total population, Number of infections, recoveries and deaths match that of the state. Hence we can vouch that the undetected cases also match the number of undetected cases in the state. When quarantining the detected cases is followed diligently, the cases in the unquarantined compartments become the undetected ones. Hence we can show with certainty that the undetected+confirmed cases make up the total estimated cases.

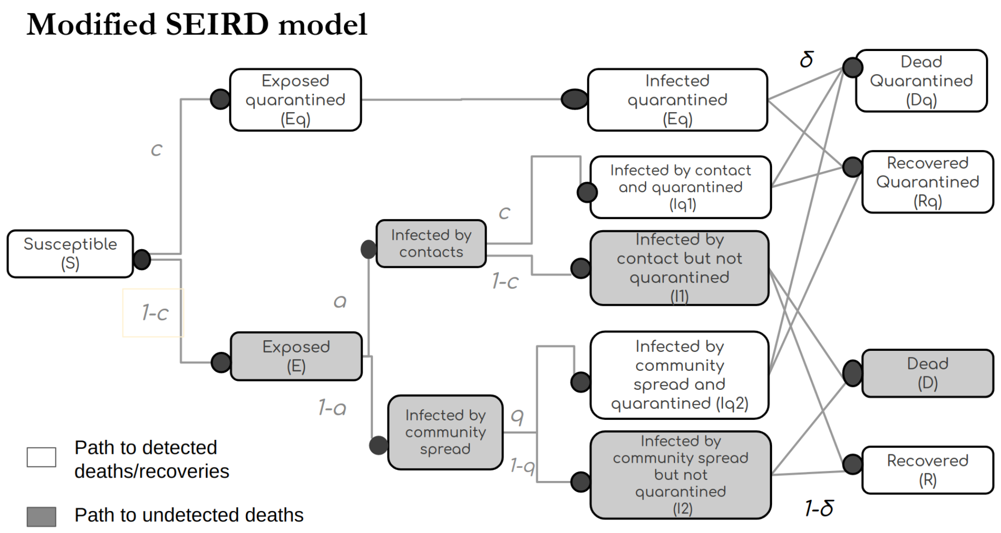

Figure 1 : This figure in the manuscript is an adaptation of Figure 1 from [4]. It shows the flow of population in compartments that eventually ends up as undetected deaths. From here it is evident that detected cases and the undetected cases together make up for the total estimated cases.

Response to reviewer #1, Q4
I agree with the reviewer's statement. The authors claim ranking needs sufficient data, like death rates; however, their ratio metric also relies on reporting of numbers of deaths (which as noted below may be underreported). Would the same issue apply?

Studies in [1] are performed by employing clustering algorithms on the actual ground data (from the period of March 2020 to May 2021). Hence to be able to characterize and cluster the states or districts, sufficient cases and deaths need to be reported. But as we have indicated in our answer, we use a simulator to predict future cases using the model parameters fit to the present day. This will help us analyze the growth of the epidemic in the near future. Therefore the metric D_ratio is computed from the estimated numbers of cases and deaths instead of actual reported numbers. This ensures that the problem of underreporting doesn't come in the way of the predictions made using the metric.

Response to reviewer #2, Overall statement
I agree as well with this reviewer, adding social and economic assumptions must also be considered.

These results from this paper become important in a scenario as is prevalent currently wherein the amount of testing has come down drastically and contact tracing has become virtually nil. Under these circumstances if an epidemic were to start due to potent imported contagious strain or by any other means, our results show that it will be quite a while before the epidemic is actually caught using 7 day rolling average of daily new cases or active cases. On the other hand, just tracking the proposed metric D_ratio having a very low cost of compliance is a lot more feasible and can catch an upcoming epidemic wave early. Since our metric is specially sensitive at low values of 'c'(infections caught from contact tracing) and 'q'(infections caught from random testing), it is an ideal indicator of an epidemic wave because it imposes least socio economic cost but keeps the surveillance robust. For example, one could put a threshold on the D_ratio and escalate within the administration when it hits the threshold. The main advantage of this metric is that it doesn't force people to get tested, doesn't impose unnecessary lockdowns, doesn't disrupt the economics of the state or the country. Hence this scheme is better than tracking 7 day rolling averages of daily new cases or active cases in predicting an epidemic wave in scenarios described above.

Response to reviewer #2, Q7
How do the author select settings where their response statement is true "growth of epidemic in a particular state where there is less variance in the underlying parameters"? What is their measure and threshold for variance? And could they please confirm or list the underlying parameters in question in their response (are they referring to beta, c, q,

etc. as noted in the next sentence)? To note, Divino are referring to projections of hospitalizations, but the authors refer to hospitalization data – do they mean projections/estimates or reported hospitalizations (to which they refer to as 'real' data)? Please be clear in the response, otherwise, it is confusing to assess the validity of the response.

We would like to clarify that the term variance here is used to convey the diversity seen in the model parameters and not in the statistical sense.The underlying parameters characterizing the growth of the epidemic are found to be varying with various geographical locations. This can be seen from the model parameters (beta, c-contact tracing, q-self reporting, population influx etc) configured to fit the model to the real cases in Delhi, UP and Karnataka as shown below (insert model param data) . This reveals that the dynamics within the country can be different. Hence comparing growths in different countries seems to be chaotic. The more localized the study the more reliable the predictions. Hence we have shown the effects of D_ratio on cases in multiple states. Although we are using the data from different states to demonstrate the utility of the metric, It can be used on further smaller set populations like towns and cities to make suitable administrative interventions.

We would like to clarify that we are using only the number of deaths estimated by the simulator for all our computations and are not using real data. We consider Death as a means by which the number of infections drains from the population pool and hence we have performed our analysis using the number of deaths. But in the real world scenario, it would be valuable to consider Hospitalization numbers for computing the metric (in which case D_ratio is replaced by the H_ratio) , as it would imply the same scenario where there is a drainage of population from the infection compartment that occurs much earlier than death. This highlights the utility of the metric as an early indicator that still predicts the course of the pandemic without actually risking a death.

Response to reviewer #2, Q8
To the authors' point (1) why is reinfection not modelled? This is not representative of the real-world situation. Is it for simplicity sake? If so, please state this in the response and reflect this in the manuscript if this has not been done. Also, please state this as a limitation if not done, and provide a hypothesis as to how this might affect projections. Not accounting for the effect of vaccinations and viral variant are major limitations. The authors must defend this choice and clearly show the effect of these limiting assumptions if this method is to be published. This should almost be mentioned in the abstract, so readers can assess the gravity.

This model doesn't consider reinfections and vaccinations for the sake of simplicity and this could be a limitation of the model.These limitations being acknowledged, parameterizing the model with respect to specific viral strains, their transmissibility and immunological properties would make it more robust and more representational of the real world scenario. But as highlighted in the Discussions, most of these parameters are understood only after sufficient time into the epidemic. Hence we have limited the model parameters to be simple so as to allow

even administrators who are non experts in epidemiology to be able to tweak them and introduce appropriate interventions much earlier in the epidemic.

Response to reviewer #2, Q11
For the table labelled as Figure 4 (it is really a table and should be labelled as such) in the table header row please label the units for the columns (days), and explain the variables in the row headers in the figure (table) caption. Also describe the shading. In the paragraph below the caption, the authors state 'when the mean infection times are very high', how high is very high? They note 'mean', but this is not specified in the table nor the caption (nor is 'average'), as well as mean latency times – latency is not shown in the table in Figure 4 – where/how does this come into play here? Please be clear.

Thank you very much for your suggestions. We have made the necessary changes to the table headings. Here mean latency period denotes the period between an individual contracting the disease and to the time one is capable of spreading the infection to others in the population. Mean infection period denotes the duration of the infection in an individual, i.e from the time one tests positive for the infection to the time one tests negative. These values here are represented as model parameters that characterize the epidemic and hence are modelled as scalar values.

Consequent to your comment, we found that applying cross-correlation to determine how early the metric catches an upcoming wave yielded very highly variable results across states. Hence we went back to design a new method to capture how early each of the metrics is able to detect an upcoming wave. According to this method, the day the metric is higher than its maximum value in the last 20 days, is used to trigger an alert predicting an upcoming wave. We have found this method to be consistent across states and different metrics. Using this to assess the performance of D_ratio v/s daily new cases and active cases, we make the following observations.

**Time lag between D_ratio and number of active cases (Days)**						
**Mean latency period**	**3**	**5**	**7**	**9**	**15**	**30**
**Mean infection period**						
**6**	40	39	37	35	30	23
**9**	70	75	39	37	34	26
**15**	50	70	77	83	98	30
**30**	45	56	73	79	100	NA

Table 1 : The table shows the number of days by which D_ratio lags(detects earlier) daily active cases in predicting the wave when all other model parameters are kept constant. The columns represent the mean latency period and the rows represent the mean infection period. It is seen

that D_ratio consistently performs better than daily active cases in the range of latency and infection periods. The cells with NA represent cases when daily active cases failed to predict a wave.

Time lag between D_ratio and number of daily new cases (Days)						
**Mean latency period**	**3**	**5**	**7**	**9**	**15**	**30**
**Mean infection period**						
**6**	39	38	36	34	30	23
**9**	NA	40	38	36	33	26
**15**	NA	NA	NA	39	35	28
**30**	NA	NA	NA	NA	NA	30

Table 2 : The table shows the number of days by which D_ratio lags(detects earlier) daily new cases in predicting the wave when all other model parameters are kept constant. The columns represent the mean latency period and the rows represent the mean infection period. It is seen that D_ratio consistently performs better than daily new cases in the range of latency and infection periods.The cells with NA represent cases when daily new cases failed to predict a wave.

This has been reported in the results section in the revised manuscript.

ADDITIONAL COMMENTS
MAJOR COMMENTS

1. Can the authors speak to the issue of underreported COVID-19-related deaths early in the manuscript?

Thank you very much for the suggestion. We have reorganized the introduction to incorporate your feedback.

2. The Introduction leads with a detailing of compartmental models. This seems to be a lost opportunity, whereby the authors should set the scene for the focus of this study. Extensive detailing of compartmental models should be reserved for the Methods.

Thank you very much for the feedback. We have moved the details of compartmental models to the methods and have reorganized the introduction to describe the motivation for our study.

INTRODUCTION

India witnessed a huge part of the population affected by the COVID-19 virus in 3 waves from March 2020 to March 2022 leading to a major crisis in saving lives, handling healthcare resources as well as suffering economic losses. Several administrative measures like lockdowns, social distancing, quarantining etc were implemented to curb the spread of these infections. These strategies should be invoked appropriately based on insights from epidemiological models. In the recent outbreak of COVID-19 parameters like the reproductive number (R0), serial interval, incubation period, transmissible period, proportion of confirmed cases in the population , proportion of deaths in critical cases and beta are some salient parameters used for modeling .While these are some epidemiological parameters, COVID-19 is also characterized by many biological parameters like variance in transmissibility period and immunological strength across different age groups and viral strains, effect of vaccination etc .But these parameters are hard to study in an ongoing epidemic. The authors highlight the complications of fitting the model to reported cases, as the cases can be distributed unevenly geographically.This would mean that there would be a sizeable population having latent infections that escapes being reported. Hence the authors suggest that hospitalizations and deaths are more reliable.This way deaths and hospitalizations that get diagnosed with the infection are from the pool of latent infections thus acting as an indicator of latent infections.

The reasons for undetected cases are many. People who are asymptomatic or with limited access to healthcare (forming a large part of the population) might go undetected easily and hence the number of confirmed, recovered and deceased cases that are reported everyday can be an underestimate of the true figures. Further insufficient testing, contact tracing or quarantining may contribute to this inefficiency. Very few techniques like PCR and antibody tests are used to estimate the number undetected infections in a particular population.These techniques are highly randomized and can only estimate the undetected cases at specific time point making the data very sparsely sampled.

Metrics like number of Active cases and deaths are looked at for studying the case growth patterns in different Indian states and ranking them based on severity of the spread. A good amount of time has elapsed before some insights can be drawn from these metrics. Furuse ,2021, describes the correlation between reproductive rate and test positivity rates showing how testing failed to catch up with the reproductive rate. Further contact tracing is also biased to the serial interval in symptomatic people. Despite being capable of predicting an impending wave, these metrics are sensitive to testing strategies and cooperation by the population in different states making them unreliable.

Amidst all the chaos of asymptotic cases, under-reporting etc, death is an unmissable event in an epidemic. The number of deaths in an epidemic provides a realistic picture of the extent of infection still latent in the population. While it is intuitively recognized that detection of infection postmortem or at first presentation at intensive care units are an indicator of the gravity of the situation, this has never been quantified or studied as a measure or indicator of relevant quantities. Current reporting practices do not capture 'infections detected postmortem' or 'previously undetected infections presenting at ICU' separately from the detected infections. We

propose that these metrics are vital indications of the health of a community or society and can be an early indicator of an emerging epidemic wave.

Modified SEIRD model describes most of the above parameters in its model and uses the epidemic simulator as a tool for administration and governance. It uses compartments in two parallel tracks representing the quarantined and unquarantined population which makes it relevant in the days of COVID-19 pandemic. This model demonstrated the utility of using simulators for predicting the number of infections in both the quarantined and unquarantined arms of the model in the normal course and in response to a variety of administrative actions, thereby estimating the effectiveness of administration reforms. These models empower the policy makers and health workers to understand the model parameters better and take necessary and timely interventions. It is well known that testing helps flatten the curve while undetected and latent cases spur on a full blown epidemic. The epidemic simulations helped to quantify this effect as a measure of inefficiency.

Using the epidemic simulator described in , we demonstrate the utility of such metrics in early detection of an epidemic wave that is hastened or exacerbated by inefficiency on account of insufficient testing, tracing or containment measures. We use the symmetry in the two arms of the model along with the fact that death of either kind is unmissable, to estimate the inefficiency (or latent spread) of an epidemic. We show here that recording the number of 'postmortem detection of infection' as a proportion of total deaths can be a good metric. The ratio of 'previously undetected infections at hospitalization' to total hospitalizations would also serve a similar purpose. We hereby make a case for modifying the Standard Operating Procedures to make this book keeping possible.

MINOR COMMENTS

1. The word choice 'hitherto' in the title and abstract and 'flatter' in the abstract may not be appropriate choices.

Thank you for suggesting these changes. We have incorporated them in the revised manuscript.

2. In the abstract, is the drop in efficiency specific to any point in the pathway (e.g., successful treatment) or at any point in the pathway?

Here we refer to the efficiency of administrative interventions that can alter the dynamics of the epidemic like the extent of contact tracing and testing and lockdowns that can bring down the beta. Since these interventions target the susceptible and exposed populations, the effect of drop in efficiency impinges on the transitions from Susceptible →Exposed→Infected thereby precipitating a wave.

3. The last sentence of the abstract, 'Further hospitals may need to be mandated to distinctly register deaths/hospitalizations due to previously undetected infections.', first,

a comma should be inserted after ‘Further’. Second, will it be possible for hospitals to be mandated to register the deaths/hospitalizations ratio? In which setting or settings would this be possible? Third, it is unclear and should be even in the abstract, how any proportion of this ratio is due to undetected infections? If infections were detected, is the point the authors are making is that deaths and hospitalizations could be prevented through ambulatory treatment? This needs to be clarified.

Thank you very much for pointing out the missing comma. We have added it in the revised manuscript.
We would like to clarify that we propose that hospitals must be mandated to screen all patients seeking admission for treatment of severe symptoms and report the number of COVID-19 and non COVID-19 hospitalizations and not the ratio in itself. By doing so, the epidemiologists, modelers and administrative bodies monitoring the epidemic can use this reported data in further calculating our proposed D_ratio metric by collating data obtained through other testing strategies. This reporting would not pose any additional logistical problems to the hospitals and should be possible under all settings. The infections that get surfaced from here would contribute to our metric and help in predicting the wave, and the likely number of cases in the peak thereby providing enough time to plan mitigation measures.

4. From the introduction, this sentence should be reworded, ‘Hence the authors say that models fit to hospitalizations and deaths are more reliable’, particularly the word ‘say’. Also, how are these model more reliable, with respect to what process (calibration) and/or outcome (forecasting for which outcome indicator)? Be more specific.

Thank you for the suggestion. We have rephrased the sentence in the manuscript to “Hence the authors suggest that hospitalizations and deaths are more reliable.” Just by fitting the model to confirmed cases, one might still be far away from the real number of infections in a population owing to testing strategies and under-reporting of cases. This would imply unrealistic model parameters like beta and reproductive numbers to estimate the duration of the epidemic. Only when the deaths and hospitalizations are also considered, we get closer to actual reproductive number as an outcome indicator

MINOR FORMATTING

1. The first sentence of the Introduction, spaces are missing after commas ‘SIS,SIR,SEIR’.

Thank you for the suggestion. We have corrected it in the manuscript.

2. Make use of ‘covid-19’ and ‘covid19’ consistent throughout the manuscript.

Thank you for the suggestion. We have corrected the mistake and replaced it with COVID-19 everywhere.

REFERENCES

1. Raj A, Bhattacharyya P, Gupta GR. Clusters of COVID-19 indicators in India: Characterization, correspondence and change analysis. SN Comput Sci. 2022;3: 210. doi:10.1007/s42979-022-01083-3
